# Special Issue: HPV and HPV Vaccines

**DOI:** 10.3390/v14020274

**Published:** 2022-01-28

**Authors:** Merilyn Hibma

**Affiliations:** Department of Pathology, Dunedin School of Medicine, University of Otago, Dunedin 9054, New Zealand; merilyn.hibma@otago.ac.nz

Our fundamental understanding of papillomaviruses and their interactions with their host, including their role in cancer and how the immune system responds to them, has made the elimination of cervical cancer a realistic global health goal. Articles and reviews in this Special Issue explore aspects of papillomavirus infection and latency, immunity, vaccination and self-sampling that inform this global health goal of elimination. This Special Issue includes excellent comprehensive reviews on the role of papillomavirus in carcinogenesis and on the viral life cycle in the cervix. Papillomavirus is increasingly associated with head and neck cancers and has been linked to the causality of cutaneous squamous cell carcinoma. This Special Issue includes reviews and original articles that extend our understanding of papillomavirus carcinogenesis at these sites. The thought-provoking review by Zheng et al. discusses viral elimination through the use of anti-viral therapies, highlighting a gap in our armoury—therapeutic approaches, either through drugs or immunisation, that can eliminate active and latent infection. The various contributions in this Special Issue highlight the need to continue to advance our understanding of papillomavirus and its interaction with its host, and how the host responds to it, so that we are able to continue to improve and refine our health interventions against it. I sincerely thank all authors who have contributed to this Special Issue, “HPV and HPV vaccines”.

